# The meaning of comfort measures only order sets for hospital-based palliative care providers

**DOI:** 10.1080/17482631.2021.2015058

**Published:** 2021-12-14

**Authors:** Suzanne S. Dickerson, Siri GuruNam Khalsa, Kathleen McBroom, Dianne White, Mary Ann Meeker

**Affiliations:** a University at Buffalo, State University of New York, School of Nursing, Buffalo, New York, USA; b University of New Mexico, College of Nursing, Albuquerque New Mexico, USA; c Seattle University, College of Nursing, Seattle, Washington, USA

**Keywords:** Phenomenology, hermeneutics, dying, palliative care, terminal care, end of life

## Abstract

**Purpose:**

*Comfort Measures Only* (CMO) is a label commonly used in the USA that guides the care of a hospitalized patient who is likely to die. The CMO label has unclear and inconsistent meaning, calling to question the experiences and practices of hospital-basedalliative care providers. The purpose of this study was to understand the meaning of CMO as experienced by hospital-based palliative care providers.

**Methods:**

Using hermeneutic phenomenological research, we investigated eight palliative care experts’ common experiences and shared practices of using CMO order sets in their hospital work settings. Data were collected through individual face-to-face interviews, and were analysed by an interpretive team.

**Results:**

Four related themes and one constitutive pattern of “Dealing with Dying” reflect the meaning of comfort-measures-only practices. The themes are: comfort care as morphine drip; enacting a traditional binary pattern of care: all or nothing; supporting patient and family at end of life vs. CMO; and evolving culture—a better way to care for the dying.

**Conclusion:**

Palliative care providers and non-palliative clinicians understood and practiced end of life care in sharply different ways with dying in hospital settings, raising new questions that analyse, modify and extend extant knowledge.

## Introduction

Over the past several decades, palliative care clinicians in the USA have endeavoured to ameliorate the pain and suffering experienced by patients dying of advanced illness. More recently, they have attempted to disseminate the knowledge and skills for expert symptom management to a broad range of healthcare providers to meet increasing needs for high quality end-of-life care (Bailey et al., 2014). *Comfort Measures Only* (CMO) is a care plan that includes physician orders that address patient’s potential bodily symptoms of discomfort that may be implemented when curative treatment has been stopped and death is expected. A search of the literature revealed that the phenomenon of *CMO*, though identified as potentially problematic, has been the subject of only limited empirical investigation and requires investigation into the meaning of CMO as it is enacted in a hospital setting.

Though commonly used, the label of CMO has unclear and inconsistent meaning across settings and providers (Do, 2014; Zanartu & Matti-Orozco, 2013). In a retrospective chart review, Walker and colleagues (Walker et al., 2011) examined differences in care for 40 hospitalized patients with and without a CMO order in place prior to death. Those with CMO orders were more likely to have a *Do Not Resuscitate* order and a family meeting. Additionally, they had more opioid analgesia orders and less utilization of laboratory tests and antibiotics. In a survey of 176 internal medicine physicians regarding their practices related to CMO (Zanartu & Matti-Orozco, 2013), researchers found no consensus related to timing of CMO orders, use of respiratory support interventions, use of antibiotics, or transfer to a higher level of care. Though a majority of participating physicians would include only oral intake in the category of nutrition/hydration, others would use intravenous fluids, tube feedings, and three reported they would include artificial parenteral nutrition. A majority would not perform laboratory tests. Nearly half of the respondents believed opioids should be used more aggressively in situations of CMO. These findings reflect confusion and diverse practices surrounding CMO.

We undertook this study of the phenomenon of *comfort measures only* prompted by anecdotal evidence from family member narratives of end-of life (EOL) care in hospital settings, especially surrounding use of “morphine drips.” Because of the critical role of prescribing clinicians in ordering CMO and referral to palliative care, hospital-based palliative care providers’ experiences and practices of how CMO order sets are enacted was sought to understand the meaning of CMO in a hospital context. Therefore, our purpose was to understand the common meanings and shared practices of palliative care providers implementing CMO in their work settings in managing end-of-life (EOL) care. In addition, we sought to gain an understanding of common concerns that call into question the practices of CMO in the hospital setting as experience by palliative care providers.

## Methods

Hermeneutic phenomenological research is an approach to find meaning and deep understanding of phenomena that occur in the world as understood through an in-depth analysis of experience, as lived (Dibley et al., 2020). The philosophy of Heidegger underpins this way of thinking and attunement to the everyday language of experiences of phenomenon that is used during data collection and analysis (Dibley et al., 2020; Heidegger, 1962). Heidegger posits that we are self-interpreting beings that are influenced by taken-for-granted meanings in our lives that come from historical, temporal and cultural meanings that are intelligible through language (Heidegger, 1962). Meaning resides in the context of the experience, thus, the interpretation becomes a rendering of explicit understanding (Palmer, 1969). p 134. Thus, during analysis of participant stories, we uncover these meanings that lend insight into understanding of our world as it shows itself in the context of possibilities and constrains (Chapter 7, p 114–132). (Dibley et al., 2020). This way of thinking informs the approach to the methods of hermeneutic phenomenology as used by other researchers such a Benner (Patricia, 2004) and Ironside (Ironside, 2005), so that researchers attune to the meanings human experience of life events—such as implementing CMO as a phenomenon of study. In-depth analysis of common experiences and often-overlooked-practices generates understanding to inform practice by raising new questions that analyse, modify and extend extant knowledge of CMO and EOL care (Vandermause, 2008).

During data collection the researcher comports him/herself as an instrument, translator and questioner, and with the participant(s), co-creates an opening for new thinking and possibilities to emerge (Vandermause, 2008). Hermeneutic phenomenological researchers therefore acknowledge that background practices (including past experiences, present meanings and future potentialities of meanings of experiences) are revealed in the interpretation of the story through the language of experiences. Therefore, interviews of the experiences of palliative care experts provide intelligible meaning through language for interpretation to illuminate life questions and future possibilities, as well as, the taken-for-granted situatedness in the health care system.

A purposeful sample of palliative care experts was recruited by means of an invitation via email following ethical approval by the University at Buffalo’s Institutional Review Board for the Protection of Human Subjects. All members of palliative care consultation teams in local hospitals who were qualified to prescribe care were invited. Eight of eleven invited clinicians participated, three were unavailable. All participants were experienced in palliative care, and at the time of interview, were working in hospital-based palliative care in several different institutions in one urban area. Following provision of an information sheet and verbal informed consent, interviews were conducted by the senior author (MM), an experienced qualitative researcher. Each interview was conducted in-person in the researcher’s university office or the participant’s private office within the healthcare setting. Each participant was invited to talk about their experiences related to past CMO implementation in their hospital followed by suggestions for improving EOL care. Interviews were audio recorded, professionally transcribed and verified by the researcher, generating 88 pages of text for analysis. The semi-structured individual face-to-face interviews lasted from 60–120 minutes, with eight palliative care specialists, including four physicians, two nurse practitioners, one physician’s assistant, and one clinical nurse specialist. Six participants were female. Age ranged from 31 to 71 years (M = 54). Participants reported 2 to 34 years (M = 19) of experience in palliative care. All held speciality certification in palliative care.

### Analysis

The five-member analysis team included an expert in hermeneutic phenomenology, an expert in qualitative methods, 3 content experts who were PhD students trained in the method, who interpreted the interview transcripts in a reflective process that followed iterative steps (Vandermause, 2008). The team read each transcript to gain an overall understanding of the participants’ stories. During team meetings, they shared summaries and identified themes from each interview with verbatim excerpts. Discussions and dialogue refined the themes and interpretations. Any discrepancies were discussed, and team members returned to the text for clarification of the participants voice and reaching consensus. As each text was read, this refinement continued, and each text was compared to the previous texts until the team, through the ongoing dialogue with the text in a hermeneutic circular process, explicated a nuanced understanding. The transcribed texts were used for interpretation of the participants’ experiences and team’s interpretation of the phenomenon. To remain open, the researcher must be able to relax (not eliminate) pre-understandings and thoughts about what is expected to be found, and have a willingness to hear all perspectives, engage in discussion, be able to experience the readiness to the question, and an openness that allows dialogue for unanticipated understandings to show themselves. This “holding open” is part of the comportment of the researcher in the interpretations, anticipating but not without question. Each member of the interpretive team shared their preunderstandings and potential themes. A reflexive journal was written during analysis that affords openness to keep preconceptions in check and focus on the phenomenon in the narratives of the participants (chapter 8 pp-136156. (Dibley et al., 2020)

Interpretation is iterative, circular, reflective and reflexive, dynamically engaging in thinking that arrives at a temporal understanding of the situation and reveals and extends understanding of human situations as they are experienced. The interpreters reflect on their explicit presuppositions (preunderstandings), creating an engaged openness and closeness with the text to understand the human situation as lived (Dibley et al., 2020). This preunderstanding involved reflections by all members of the interpretive team, who are nurses with experiences caring for patients at the end of life. Two members are experts in palliative care research. Although in hermeneutics the assumption is that no single correct interpretation is possible, the interpretations are focused and reflective of new possibilities for practice. The related themes (present in some transcripts) and constitutive pattern (present across all transcripts and supported by the relational themes) were examined by the team for coherence and comprehensiveness (Dibley et al., 2020).

Rigour was maintained throughout the study by use of DeWitt and Ploeg’s (De Witt & Ploeg, 2006) framework of balanced integration, openness, concreteness, resonance and actualization. *Balanced integration* was achieved in the study findings by a balance between the voice of the participants and the researchers’ interpretations, as explicated in the themes and constitutive pattern, use both verbatim excerpts and interpretive explanations, *Openness* was achieved by a process of auditing the interpretive decisions, returning to the text for verification and consensus in the group analysis as recorded in reflexive journaling regarding preunderstandings and what shows itself in the narrative. *Concreteness* was achieved in the findings by providing context to situate the reader in the phenomenon by using verbatim examples that resonate with life experiences. *Resonance* is achieved when the reader has an intuitive grasp of the meaning of the study through reading the findings and verbatim examples. *Actualization* addresses future resonance of study findings in future readings of the interpretation.

## Results

Four related themes and one constitutive pattern of “Dealing with Dying” reflect the meaning of comfort measures only practices in the experiences of palliative care experts.

### Theme one: comfort care as morphine drip

CMO order sets were developed by administrative teams including pharmacological focused experts to provide medical orders for patients who were dying. Participants told stories of when curative efforts were no longer meaningful, as a central feature of the order set, comfort care for a hospitalized patient historically centred on a continuous intravenous infusion of morphine. This standard intervention seemed to have long-term, deep-seated roots within the hospital culture, becoming the standard “treatment” for dying. While participants acknowledged that a morphine infusion could be useful in situations of severe uncontrolled pain, it had come to be implemented reflexively rather than thoughtfully and appropriately. For example, one participant related:

In this institution, there’s a default to ‘you’re comfort care; now let’s hang some morphine.’ It’s not ‘let’s assess’ …. I actually got asked by the nurse practitioner taking care of a woman on another service, who was happily watching Golden Girls on the TV, to start a morphine drip, to which I replied, ‘why on earth would I start a morphine drip on someone who’s watching television?’ I got told, ‘well, she’s comfort care now.’

Another participant reported having the same experience when questioning a patient’s need for a morphine drip and being told, “Well, because they’re comfort care.”

As a morphine infusion became the emblem of EOL care, it also tended to be used as the only available treatment. Prior to implementation of palliative care teams, in the context of providers not educated to recognize and respond to a diversity of discomforts common during dying, all discomforts were addressed by increasing the infusion rate of the morphine. Nurses responded to any expression of patient (or, sometimes, family) distress by increasing the rate of the drip. Medical orders encompassed titrating to a maximum dose, or sometimes opened-ended “titrate to comfort.” Everything that disturbed a patient was “oh, let’s increase the drip”. Historically hospital staff’s reflexive use of morphine drip denoted a focus on the everyday practices of routine biomedical care with lack of focus on the dying experience of the patients and their families.

Participants related that use of a morphine drip was sometimes distressing to family members as it could stop communication with the patient. As one described:

Families have come to me and said my sister was awake this morning talking to me, but she stopped talking when they started the morphine drip. She’s no longer able to arouse.

Another participant described how the morphine drip was part of the prevailing culture:

It was the culture …. it’s a mindset that the nurses have ‐‐ ‘They’re comfort care; they need a drip’ ‐‐ without thinking about it. I remember one little guy in particular who was in his 80s, and he was dying, but he did not need a morphine drip. He was awake and alert and communicating with his family. They made the decision to go for comfort care, and they started the drip.

Participants sometimes likened the practice of using morphine drips as comfort care to camouflaging euthanasia. Participant experiences revealed suggestions—ranging from subtle to overt—that increasing the rate of the morphine drip could help speed up dying. Both providers and family members were sometimes heard to question why dying was “taking so long”. From physicians, there was sometimes pressure because others needed the bed. One participant was told, “I’ve got four other patients that need to get in here and need to get treatment or they’ll die, and he’s taking up a bed”. From exhausted family members, particularly if they had been led to expect that death was imminent, there was sometimes a direct request to speed up dying. As reported by one nurse practitioner, “It’s like, ‘Why is this taking so long? Hurry. Speed this up’”. A physician reported that sometimes families would ask directly, “Yeah, add the morphine so it’ll happen quicker, we’re all tired, can’t we make this go faster, what are we waiting for, we know they’re dying; what are you doing?”.

In another setting, the provider sensed an unspoken understanding about hastening death:

I worry that may be an intention-an unspoken intention, but an intention. It’s been hinted by various providers that the family doesn’t disagree with this approach. In other words, it’s okay for us to bump our patient off because nobody’s going to complain, which I find objectionable.

Prior to palliative experts being fully utilized in their settings, over time, the CMO order resulted in the reflexive use of a morphine drip as the sole comfort measure to manage symptoms, yet served to reduce patient communication with family members and may have hastened the dying process. The next theme considers the manner of enacting CMO.

### Theme two: enacting the traditional binary pattern of care: all or nothing

In describing how CMO was enacted in their institutions, palliative experts focused on practices surrounding transition to comfort-focused care as representing two sharply distinct approaches to care, which they characterized as “full-bore” and “full-stop.” As described by participants, medical providers would rush severely ill patients to the intensive care units where all options were explored to keep the patient alive. The providers were going “full bore” to fix the body- until they ran out of possible interventions. This practice revealed a mechanistic metaphor of body as machine as compared to body as person. One participant spoke of medical providers “fascination with rescue”. Seeing the body metaphorically as a machine meant that providers believed that “nothing more could be done” when the body could no longer be fixed using the tools of medicine. The existential being of the patient was ignored. This view was fundamental to how subsequent decisions were made, as this participant reported:

Sometimes what I’ll hear is ‘this is a salvageable patient,’ like we’re talking about human beings that are salvageable instead of talking about what the goals are of that patient and family.

Within the medical model described in palliative providers’ stories of patient care, the inability to cure or rescue, because that was the pre-eminent and most valued goal, reflexively became stopping all interventions. Thus, comfort care became not doing or a “full stop”. As one participant reported about the institutional mindset, “There’s still very much that message of comfort care, stop everything”. Historically, the focus on what was not to be done at the end of life began with the emergence of DNR (Do Not Resuscitate) orders. Thus, palliative or comfort care was enacted as refraining from interventions, which our participants found problematic as palliative care providers. Comfort care has been “dumbed down to what we won’t do rather than what we could proactively offer”. Another participant, noting the contrast between the thoughtful practice of palliative care and the “stop everything” mentality, remarked that palliative care providers were “not [going] to drop people off the face of the comfort care cliff”.

Another participant conveyed frustration with how CMO was enacted:

The other objection I have to the term is a philosophical one, because I’ve never wanted palliative care, or particularly hospice care, to be defined by what we don’t do and to be defined by services we don’t offer, measures we won’t undertake. I would much rather have an ongoing, dynamic dialogue of what is reasonable in a plan of care given the patient’s goals, hopes, objectives, and the reality of the circumstances in which they find themselves.

Palliative care providers described how family members experienced distress when the plan of care shifted to a focus on what would not be done. One participant described the family’s perspective as saying, “I don’t want you to push my family member into the corner just because I say they’re comfort care. I want you to still do things for them”. Another participant described:

What [families] they’ve heard from the team is comfort care means we’re going to stop this, and stop that, and the family backs away and says ‘no, I don’t want you to stop everything.’ It’s this *we can’t do anything more* concept.

The participants told of patients and families feeling abandoned, which was consistent with the “stop everything” mentality of caring for a dying person, and the failure to discern any value inherent in the experience of dying or losing a loved one. Participants described how a strongly cure-focused approach to care could lead to devaluing and marginalizing a dying patient. In one hospital, an upper level manager, when asked by a nursing supervisor for additional staff, “questioned, ‘why do you need more staff for that—they’re dying’”.

In some situations, when cure was no longer a goal, cure-focused providers ended any relationship with the patient, suddenly leaving patient and family care to palliative providers to deal with the ensuing abandonment. While the palliative care providers in this study expressed willingness to assist anyone who needed their care, they criticized physicians who referred to the patient to them [palliative care team] and then ceased all contact with a patient for whom they had been caring for a long time. The patient and family had no previous relationship with the palliative providers. This was distressing to palliative providers especially when it happened close to death rather than earlier in the disease trajectory. One participant described a 21-year-old patient who chose to relinquish curative care and the response of the physician following referral to palliative care, “He wouldn’t come back to answer more questions; he just washed his hands: ‘I’m done’”.

### Theme three: supporting patient and family at end of life (vs. comfort measures only)

Participants reported that the creation of CMO standard orders was prompted by the need for non-palliative providers to have guidance in managing distressing symptoms commonly arising at end of life. Yet, rote implementation of comfort care orders was seen as antithetical to good care. One participant, when asked if the institution where he worked had a standard order set for comfort care, replied, “When we got them there, yes …. I spent a lot of time trying to get rid of them”. All participants agreed that having a standard order set could be problematic, tending to promote unthinking and “cookie-cutter” application to care. One nurse practitioner advised, “Use it [CMO standard orders] as a stepping stone for your own growth, then it’s a good thing, but I think most people in a busy day use it as a crutch rather than a stepping stone”.

When clinicians employed a CMO approach, they frequently failed to address sensitively the patient’s needs for ongoing symptom management. Participants talked extensively about observing other providers’ failure to recognize delirium and consequent mismanagement with morphine rather than with a more appropriate drug. As one reflected:

Not recognizing that something might be delirium or something else that was inappropriately treated with an opioid, and so they just go for everything. Any time the patient makes a peep, moves, grimaces, is restless, they say, ‘They’re in pain. Give them [more morphine].

Participants caring for patients and families at the end of life focused on symptoms of the corporeal body and at times focused on the lived body of the patient, who was still interacting and living in their changing world.

### Theme four: evolving culture—a better way to care for the dying

Study participants recognized that the culture of EOL care was slowly being changed by their involvement. They experienced considerable frustration with perceived inappropriate care, but also saw what they viewed as some positive changes. As one physician described the changes over time:

They [physicians from some of the other services] started using less and less of the comfort measures [order set], but would call us [palliative specialists] earlier …. So, the result is when a patient was deemed end-of-life care, instead of starting a drip, they would call us and say help us out here.

While far from complete, this evolving shift in culture, according to our participants, involved moving from “comfort measures” as a shorthand label that only ostensibly carried shared meaning, to the values, expertise, and interdisciplinary teamwork of the palliative care speciality. One participant commented on how difficult such change can be:

So there is a great inertia ….To make the culture different is a very difficult thing, and so my belief is that a part of this that I’ve seen is just one stage in the evolution of this approach to end of life care, and coming in and saying we’re going to do this differently, and I paraphrase, but I’m not far off from a direct quote—‘*that’s not the way we’ve done it’* was the standard refrain.

All participants recognized that EOL care should be thoughtfully individualized rather than the binary of everything vs. nothing—or caring only for the corporeal body of the dying patient who still is living in a lived body. One participant reflected on the transition of care focused on the person:

It should just be part of the trajectory, and removing items that are no longer beneficial as you move along the trajectory vs. having this moment where now we’ll worry about how you’re feeling physically and emotionally.

Another participant sought to generate understanding in both family members and other providers that although care was changing, care was continuing. “Care continues, and care now is going to be more intense”.

All study participants concurred that clinicians from diverse specialities needed education for providing end-of-life care. As one nurse reflected, “There’s a whole lot of education that has to happen for physicians and nurses as well.” One early-career physician reported on not being prepared during medical school:

I can say as a medical student, I was not taught anything about anything. I wasn’t taught anything about palliative medicine. I wasn’t taught anything about comfort care. I wasn’t taught about how we help a patient die with dignity, nothing. We never talked about end-of-life issues at all.

Participants stories minimally reflected on the dying person’s experiences that may go beyond medical management. Examining the silences, or what was not said by participants, we note that few referred to the patient’s being or experience of living with others while approaching death or authentic “being-towards-death”. No stories of spiritual experiences or needs or facilitating acceptance of death were mentioned.

### Constitutive pattern: dealing with dying

A constitutive pattern connects the four related themes to explicate the meaning of comfort measures only care from palliative experts’ stories of enactment of CMO in hospital settings. This study informs our understanding of current trends in clinical care for hospitalized patients who are shifting from curative to supportive care at end of life. Although the label of CMO appeared to carry shared meaning, both understanding and modes of implementation varied. Initiation of an order for CMO commonly signified a discrete and dramatic shift in the approach to care. *Enacting a binary pattern of all or nothing* occurred that often was as simplistic as “stop everything” and start a “morphine drip”. In an all or nothing approach, the providers’ objectifying discourse of biomedical science, treated the patient as a corporeal body, an object that was detached from the lived-body. As Heidegger (Heidegger, 1961/1979) said “We do not ‘have’ a body; rather we are bodily … we are somebody who is alive” (p. 99). The lived-body refers to the person’s own experiences, feelings, and interpretations of his/her world, as lived. Patients nearing end-of-life experiences face a future of constriction of meaningful possibilities, yet still have the possibility of having life experiences worth living. Heidegger (Heidegger, 1962) also claims that “being-in-the-world” is always ‘being there with others.” (p. 152), thus patient’s wishes can be heard in an attentive engagement with others in a discursive context that situates and acknowledges suffering and gives meaning to the time left.

Alternatively, participants reported numerous situations in which the care decisions had a mindless, reflex quality rather than a careful, comprehensive, person-centred assessment of what would enhance comfort and what would impede it. Supportive care at the end of life was described as a focused approach to symptom management that recognized the dying patient and individualized symptom care. The evolving palliative care culture was concerned with individualized symptom management that focused on the body, yet participant stories were still less focused on the dying person’s lived experiences. Furthermore, the stories were silent on spirituality and approaches to facilitate the patient’s authenticity in being-towards-death in a way that is open and receptive to the possibility of death.

Palliative care providers were attempting to change the culture to counter the patterns of CMO practices harmful to patients and neglectful of families that still may exist. These changes call for a shift in focus from the patient as a corporeal body to an interactive person living in the world and attempting an authentic being-towards-death.

## Discussion

Our study found that with a strong interdisciplinary palliative care team in place and repeated discussions and education of the physicians and nurses by those palliative experts, some improvements in EOL care for hospitalized patients were being seen over time. Practice was gradually shifting towards contacting the palliative care team earlier in the patient’s illness trajectory. Palliative care providers were working to support the transition to a person- and family-centred culture of care, including thoughtful individualization of care, education of providers, and increasing awareness of the meaning and value of palliative care.

Our findings illuminate the pervasive influence of differing root metaphors or worldviews as they provide the framework underlying and informing professional healthcare practice. A root metaphor provides a particular philosophical lens that guides thinking and perception. Following Pepper’s model (Pepper, 1942), Lyddon (Lyddon, 1989) describes four core ways of viewing the world. Two of those, mechanism and organicism, are especially relevant for our findings. Mechanism is based on the root metaphor of *machine*, and suggests linear chains of cause and effect, wherein phenomena are understood through their parts, each of which has a distinct position and role. In the biomedical model, the body is likened to a machine, each part of which can be replaced, repaired, or strengthened. As reported above, such a metaphor can lead to thinking of a patient as “salvageable”. In contrast, the stance of palliative care aligns more closely with Pepper’s root metaphor of *organic process*. Here the emphasis is on inherent wholeness and complex and integrated processes and systems rather than on individual elements and cause-effect relationships. The patient is not limited to his or her weakening body. However, the silences observed in the participants’ narratives and omission of comments on spiritual or existential experiences of patients in their care suggest that while committed to individualized person-centred care, they, too, had been influenced by the medicalization of suffering (Davis, 2010).

Our findings regarding the lack of shared meaning for the label of CMO are validated by others (Kelemen & Groninger, 2018) as is the view of our study participants that not only is the term no longer clinically useful, but that it can be a source of harm. Our participants advocated dispensing with the shorthand label of CMO, and carefully and thoughtfully considering the patient’s unique situation and values to choose interventions that could effectively provide comfort and support and to relinquish those that burdened or were useless.

In contrast, some believe that standardized order sets have value, but need improvement (Bailey et al., 2014; Bender et al., 2017; Lau et al., 2018). In one quality improvement effort (Bender et al., 2017), researchers revised comfort care order sets of two academic medical centres with a focus on improving the use of infused opioids in intensive care settings. Others have undertaken more extensive efforts to improve care by means that include order sets, but attend comprehensively to education and implementation (Bailey et al., 2014). The path to consistently improving patient experience and outcomes has yet to be clearly identified.

In a recent philosophical critique of evidenced based care (Fearon et al., 2018), the authors felt that quantitative approaches do not provide answers to complex problems such as opioid use in palliative care, but rather reflect inherent biases of pharmaceutical grant supporters. Qualitative approaches that address the alternative research paradigms would give rise to understanding approaches to pain at end of life that reflect the patients’ varied palliative care needs.

Our findings clearly indicated that palliative care specialists and acute care providers held diverging concepts of comfort. An understanding of what patients, as well as family members, regard as important to comfort would be helpful in future studies. Coelho and colleagues (Coelho et al., 2016) employed a phenomenological approach to examine how palliative care patients defined comfort and its precursors and to generate a preliminary model for the concept, inclusive of relational and spiritual concerns. We believe more such study is needed to deepen our understanding of EOL comfort, our sensitivity to diverse patient views (Hundt, 2021), and our capacity to deliver care that engenders meaningful comfort as often as possible.

Our study adds to an understanding of the historical and cultural barriers to changing care practices. Arising from a grass-roots movement in home-based care at the end of life, palliative care has now moved into acute care settings where the challenges of effecting end-of-life culture change are more complex (Bailey et al., 2014). The “notion of home” as a goal of palliative care (Dekkers, 2009) could open a crucial dialogue in understanding the patient’s and family’s goals and interacting as being-in-the-world versus the curative view that reduces being to a corporeal body.

In our study what was revealed is the common philosophical perspective of corporeal body versus lived-body, which uncovers the taken for granted meaning of curative health care in today’s hospital systems. The underlying focus on the medical regimen and care of the body, serves to limit the person’s perspective of their being and existence focusing on the patient as a passive object with limited sense of agency and being with others (Aho, 2019).

### Limitations

This study was of hospitalized patient care in one community in the Northeastern USA, from the perspective of palliative care providers. It is unknown the perspectives of those who chose not to participate or of clinicians not trained and experienced in palliative care. Future studies need to include care recipients, both the individuals that are living with dying and their families.
